# BIN1: A Protein with Great Heart

**DOI:** 10.1371/journal.pbio.1000311

**Published:** 2010-02-16

**Authors:** Caitlin Sedwick

**Affiliations:** Freelance Science Writer, San Diego, California, United States of America

**Figure pbio-1000311-g001:**
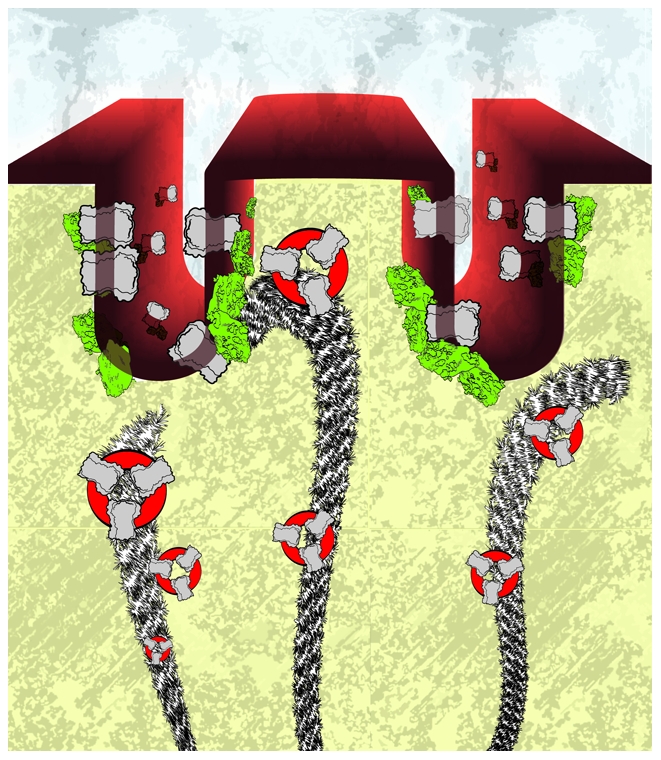
L-type calcium channels must localize to “T-tubule” membrane invaginations of heart muscle cells. New data suggest the channels are delivered by dynamic microtubule highways that tether specifically to T-tubules via the membrane curvature protein BIN1. (Image Credit: Jacob Vogan)

[Fig pbio-1000311-g001]Heart tissue is made up of a specialized type of muscle cell known as a cardiac myocyte. Like all muscle cells, cardiac myocytes contract as electrical signals propagate along their cell membrane. These signals cause a specific kind of ion channel—the L-type calcium channel—to open and let calcium ions into the cell. This initial influx of calcium in turn stimulates the release of more calcium from internal cellular stockpiles. The resulting flood of calcium allows the molecular motor myosin to begin ratcheting along the parallel bundles of actin filaments that make up muscle fibers. As the myosin ratchets, opposing filaments slide against each other, thereby creating contraction.

The L-type calcium channels that make ventricular heart muscle cell contraction possible are located specifically on specialized structures known as T-tubules. These are deep invaginations of the muscle cell's outer membrane that occur perpendicular to muscle fibers, near the sites where the cell keeps its internal calcium stockpiles. Until now, how cells manage to concentrate L-type-calcium channels at T-tubules was not understood. But, in this issue of *PLoS Biology*, Ting-Ting Hong, Robin Shaw, and their colleagues explain that L-type calcium channels are targeted to T-tubules in cardiac myocytes with the help of the protein BIN1.

BIN1 has long been recognized to play an important role in muscle cell biology. It contains a region, known as a BAR domain, that allows it to interact with and induce curvature in cellular membranes. In fact, BIN1 is known to help generate the membrane curvature needed to form T-tubules in cells derived from skeletal muscle (skeletal myocytes). However, until now, little was known of the biology of BIN1 in cardiac myocytes.

Hong et al. were intrigued by the fact that mice lacking BIN1 die before birth due to failures in their heart muscle, so they decided to explore what role BIN1 might play in cardiac myocytes. First, they examined the localization of BIN1 in adult cardiac myocytes and found that BIN1 is localized on the T-tubules. Close examination of cardiac myocyte T-tubules also revealed that the spots where BIN1 clustered together most tightly also had high levels of the L-type calcium channel Cav1.2. Therefore, the authors wondered whether BIN1 might actually be involved in helping recruit Cav1.2 to T-tubules.

To test the idea that BIN1 might recruit Cav1.2 to membranes, Hong et al. added BIN1 to HL-1 cells (an atrial heart muscle–derived cell line that normally lacks T-tubules, but does express Cav1.2). When BIN1 was expressed in these cells, it caused the formation of deep invaginations in the cell membrane—structures reminiscent of T-tubules. BIN1 clustered along these invaginations and, as in cardiac myocytes, Cav1.2 was found concentrated at the BIN1 clusters.

How does BIN1 recruit Cav1.2 to T-tubules? Existing literature suggests that after being manufactured by the cell's protein factory, many ion channels are delivered to their specific membrane destinations by following the lengthy fibers of the cell's microtubule cytoskeleton. Accordingly, Hong et al. considered the possibility that BIN1 might serve as an anchoring site for microtubules. Indeed, they found that the ends of growing microtubule fibers extend toward clusters of BIN1 protein. Furthermore, once a BIN1 cluster manages to snare the free end of a microtubule fiber, the microtubule appears glued to that spot for a protracted period, suggesting BIN1 might act as a kind of molecular landing pad for Cav1.2 delivery along microtubules.

To explore what portions of the BIN1 protein are needed for this activity, the authors created a mutant version of BIN1 that was truncated after its BAR domain. This mutant protein still induced membrane curvature and membrane invaginations when it was expressed in HL-1 cells, but Cav1.2 failed to cluster at these invaginations. Therefore, the authors concluded that the ability to recruit Cav1.2 resides in a portion of the protein distinct from its BAR domain.

Collectively, the authors' data suggest that BIN1 plays a dual role in cardiac muscle cells; not only is it needed to help generate T-tubules, but it also designates T-tubules as the appropriate site for delivery of L-type calcium channels. The significance of this is underscored by an experiment in which the authors knocked down BIN1 expression in cardiac myocytes using RNAi; in these cells, release of calcium from internal stores is significantly impaired. Because similar conditions occur in heart failure, these findings suggest that BIN1 might play a role in cardiac disease—a possibility the authors are currently investigating.

**Hong, T-T, Smyth JW, Gao D, Chu KY, Vogan JM, et al. (2010) BIN1 Localizes the L-Type Calcium Channel to Cardiac T-Tubules. doi: 10.1371/journal.pbio.1000312**

